# 

**Published:** 1882-11

**Authors:** 


CLINICS.

MONDAY.

Eye and Ear Infirmary—2.15 p. m., Ophthalmological, by Prof. Hotz.

Mercy Hospital—2 p. m., Medical, Profs. Hollister and Quine.

Rush Medical College—3 p. m., Dermatological and Venereal, by Prof. Hyde.

Woman's Medical College—2 p. m., Dermatological and Venereal, by Prof. Maynard.

TUESDAY.

Cook Co. Hospital—2 to 4 p. m., Medical and Surgical Clinics.

Mercy Hospital—2 p. m., Surgical Clinic, by Prof. Andrews.

Woman's Medical College—10 a. m., Prof. Ingals.

WEDNESDAY.

Chicago Medical College—2 p. m., Eye and Ear, by Prof. Jones.

Rush Medical College—2 p. m., Medical by Prof. Bridge; 3 p. m., Ophthalmological and Otological, by Prof. Holmes: 3 to 4 p. m., Diseases of the Chest, by Prof. Ross.

Woman's Medical College—2 p. m., Eye and Ear, by Dr. W. T. Montgomery; 3 p. m., Diseases of Children, by Prof. Chas. W. Earle.

Eye and Ear Infirmary—2.30 p. m.. Dr. E. J. Gardiner.

THURSDAY.

Chicago Medical College—2 p. m., Gynæcological, Prof. Dudley.

Rush Medical College—2 p. m., Diseases of Children, by Prof. Knox; 3 p. m., Diseases of the Nervous System, by Prof. Lyman.

Eye and Ear Infirmary—2 p. m., Ophthalmological, by Dr. Hotz.

Woman's Medical College—3 p. m., Surgical, by Prof. Owens.

College of Physicians and Surgeons—2 p. m., Medical, by Prof. S. A. McWilliams; 3 p. m., Surgical, by Prof. R. L. Rea.

FRIDAY.

Cook County Hospital—2 to 4 p. m., Medical and Surgical Clinics.

Mercy Hospital—2 p. m., Medical, by Prof. Davis.

SATURDAY.

Rush Medical College—2 p. m., Surgical, by Prof. Gunn.

Mercy Hospital—2 p. m., Surgical Clinic, by Prof. Andrews.

Chicago Medical College—3 p. m., Neurological, Prof. Jewell.

Woman's Medical College—2 p. m., Gynæcological, by Prof. Stevenson.

College of Physicians and Surgeons—2 p. m., Diseases of the Chest, by Prof. S. A. McWilliams; 3 p. m., Gynæcological, by Prof. A. Reeves Jackson.

Daily Clinics, from 2 to 4 p. m., at the Central Free Dispensary, at the South Side Dispensary and at the West Side Dispensary.

